# The first outbreak of herpes gladiatorum in Thailand: an investigation of boxing gyms in Phuket, May–August 2022

**DOI:** 10.5365/wpsar.2025.16.4.1142

**Published:** 2025-12-01

**Authors:** Suppasit Srisaeng, Kusuma Swangpun, Arriya Panchaiyaphum, Pilailuk Akkapaiboon Okada, Warodom Sornsurin, Panuwat Naraart, Thanawadee Chantian

**Affiliations:** a Field Epidemiology Training Program, Division of Epidemiology, Department of Disease Control, Ministry of Public Health, Nonthaburi, Thailand.; b Phuket Provincial Public Health Office, Ministry of Public Health, Nonthaburi, Thailand.; c National Institute of Health of Thailand, Nonthaburi, Thailand.; d Office of Disease Prevention and Control Region 11, Department of Disease Control, Ministry of Public Health, Nonthaburi, Thailand.

## Abstract

**Objective:**

The objectives of this study were to describe the characteristics of Thailand’s first reported outbreak of herpes gladiatorum in Thai-boxing gyms and to provide recommendations for reducing the risk of transmission.

**Methods:**

Hospital reports of atypical rashes appearing among Thai-boxing trainees triggered investigations at three gyms in Phuket during May–August 2022. Semistructured questionnaires were used to collect data from gym owners, trainers and trainees. Skin and blood specimens were collected for reverse transcription–polymerase chain reaction testing for herpes simplex virus type 1 (HSV-1), antibodies to HSV-1 and other pathogens; genomic sequencing was performed on culturable samples. The environmental investigation included walk-through surveys, a review of each gym’s web site, and testing of surfaces and water specimens.

**Results:**

Nine cases of herpes gladiatorum were confirmed and one was suspected, all in non-Thai trainees. Attack rates in Gyms I, II and III were 21.4%, 11.5% and 2.6%, respectively. Risk behaviours included sparring with partners who had a rash, sharing equipment and neglecting to shower before training. HSV-1 was detected on gym equipment and surfaces, and cultures from skin lesions and blood samples revealed a genomic linkage between two cases in Gym II, identified as belonging to the East Asian Clade II strain. Disinfection of equipment reduced exposure to HSV-1.

**Discussion:**

The first outbreak of herpes gladiatorum in Thailand was confirmed in 2022. Genomic sequencing suggested local transmission within Thailand, with the virus introduced independently into each gym. Skin-to-skin contact was likely the main mode of transmission; environmental findings indicated a lower risk of transmission via gym surfaces. Recommendations to prevent future outbreaks include stricter regulations for pre-fight screening and improved gym cleaning and hygiene among trainers and trainees.

Herpes gladiatorum (HG), a skin infection caused by herpes simplex virus type 1 (HSV-1), is prevalent in athletes who participate in high-contact sports such as wrestling and boxing. (*1*) The virus spreads through direct contact with lesions or mucosal secretions, and is most contagious when sores are present. (*2*) However, asymptomatic shedding, which occurs in 4–25% of cases, also facilitates transmission. (*3*)

Early, or prodromal, symptoms of HG include malaise, anorexia and fever; painful skin vesicles develop within 4–11 days following exposure, (*4*) typically on the forearm, face and neck, and most frequently on the right side of the body. HG is commonly misdiagnosed as bacterial folliculitis. (*4*, *5*) Around half of cases experience recurrent infections, which are usually milder. (*6*) Although severe complications are rare, ocular infection with HSV is a common cause of blindness in United States of America, and untreated herpes encephalitis has a 70% fatality rate. (*4*)

HSV-1 infection is relatively common. Data from 2012 suggest that two thirds (67%) of the global population younger than 50 years (3.7 billion persons) are infected with HSV-1; in South-East Asia, the proportion is 59%, or 432 million persons. (*7*) A 2019 systematic review concluded that 73% of adults in Asia have antibodies to HSV-1. (*8*)

Prior to 2022, there were no recorded outbreaks of HG in Thailand. However, the emergence of mpox in May 2022 raised surveillance awareness of rash-causing diseases. The Bamrasnaradura Infectious Diseases Institute (BIDI) notified the Division of Epidemiology at the Department of Disease Control, Ministry of Public Health, and several hospitals in Phuket notified the Provincial Public Health Office, about clusters of atypical rashes occurring in patients linked to three Thai-boxing gyms, triggering a joint investigation team to assess these gyms (Gyms I, II and III). This team, which comprised representatives from the Provincial Public Health Office, the Office of Disease Prevention and Control Region 11, and the Division of Epidemiology at the Department of Disease Control, conducted its investigations during May to August 2022. On-site investigations were conducted within 2 days of notification of the most recent case in each gym.

The aims of this study were twofold: (i) to describe the characteristics of Thailand’s first confirmed outbreak of this disease and (ii) to make recommendations to prevent and control outbreaks.

## Methods

### Study population

On-site investigations were initiated on 26 May at Gym I, 27 June at Gym II and 12 August at Gym III. A second investigation was conducted at Gym II on 7 July to allow for more detailed interviews and specimen collection. Across the three gyms, 114 of 257 individuals were interviewed and examined for the presence of a rash, including gym owners, trainers, trainees and other staff. The following case definitions were employed.

**Suspected case:** A person who trained or worked in any of the three gyms between May and August 2022 and developed a rash (i.e. macules, papules, vesicles, pustules or crusts), with or without fever, sore throat, headache, myalgia or lymphadenopathy.
**Confirmed case:** A suspected case with a positive result from a reverse transcription–polymerase chain reaction (RT–PCR) test for HSV-1.
**Risk contact:** A person who had direct skin-to-skin contact with a suspected or confirmed case during training or sparring sessions, or had used shared equipment, such as target pads, gloves or punching bags.

### Data collection

Convenience sampling was used to interview the individuals present at each gym and examine them for rashes during the on-site investigation. Individuals not present on investigation days were interviewed by telephone. Individuals reporting rash symptoms over the telephone were further assessed at the gym, their accommodation or a hospital. Ten suspected cases were identified through this process and were interviewed in detail using a semistructured questionnaire. Ten Thai trainers from Gym II were also interviewed in detail. The questionnaire covered demographic information (e.g. sex, age, nationality), medical history (e.g. date of symptom onset, underlying conditions, prior herpes infections, symptoms, treatment), exposure history (e.g. sparring with symptomatic partners) and hygiene behaviours (e.g. equipment sharing, showering before training). The owners of Gyms I and III did not consent to interviews.

### Laboratory testing

Specimens from throat swabs, lesion fluid, lesion scrapes, lesion swabs and/or blood were collected and tested at the National Institute of Health of Thailand using RT–PCR for mpox, HSV-1, HSV-2, varicella–zoster virus and *Treponema pallidum*. RT–PCR results with a cycle threshold (Ct) value of ≥ 40 cycles were classified as “not detected.” Higher Ct values typically indicated viral loads below the detection limit. Blood samples were additionally tested for HSV-1 antibodies. Genomic sequencing was performed on six culturable HSV-1 specimens from four cases. Viral genomic sequences were quality-trimmed and assembled into consensus sequences, then aligned against the HSV-1 reference genome (National Center for Biotechnology Information reference sequence for HSV-1 strain 17: NC_001806.2). (*9*, *10*) A phylogenetic tree was constructed using W-IQ-TREE version 1.6.11 with maximum likelihood analysis. (*11*) Geographical data for each isolate were overlaid onto the tree.

### Environmental study

Walk-through surveys were conducted in all three gyms to gather information relating to the facilities, sanitation measures, cleaning protocols, the use of personal protective equipment and training practices. The gyms’ web sites were reviewed to gather information about staff, facilities and training programmes. From Gym II, surface swabs and water samples from the pool, toilets and water supply were collected and sent to the National Institute of Health of Thailand for RT–PCR for HSV-1. Free chlorine levels in water samples were tested on-site.

## Results

### Descriptive study

#### Setting and context

Including owners, trainers, and adult and teenage trainees (i.e. younger than 18 years), 47 individuals had links to Gym I, 66 to Gym II and 144 to Gym III. Gyms II and III were large and offering training in mixed martial arts (MMA) as well as Thai boxing. The duration of training programmes ranged from 1 to 2 weeks for amateurs to 2 to 3 months for professional fighters. Training sessions were held twice daily, Monday to Saturday (7:00–10:00 and 16:00–18:00). Sessions typically began with a 30-minute run of 3–5 km, 10 minutes of skipping rope, and 30 minutes of stretching and shadowboxing. Morning sessions included an hour of clinching and sparring in 4–5 rounds of 3 minutes each, with 2-minute rest periods and 4–12 partner rotations, ending with 15 minutes of stretching. Evening sessions focused on bag and pad work (i.e. striking punching bags and targets held by a trainer). Saturday sessions featured Thai-boxing pre-fight ritual dancing and full-body training techniques involving frequent upper-body contact. Thai-boxing techniques and clinches are shown in **Supplementary Fig. 1**.



#### Case characteristics

**Fig. 1:**
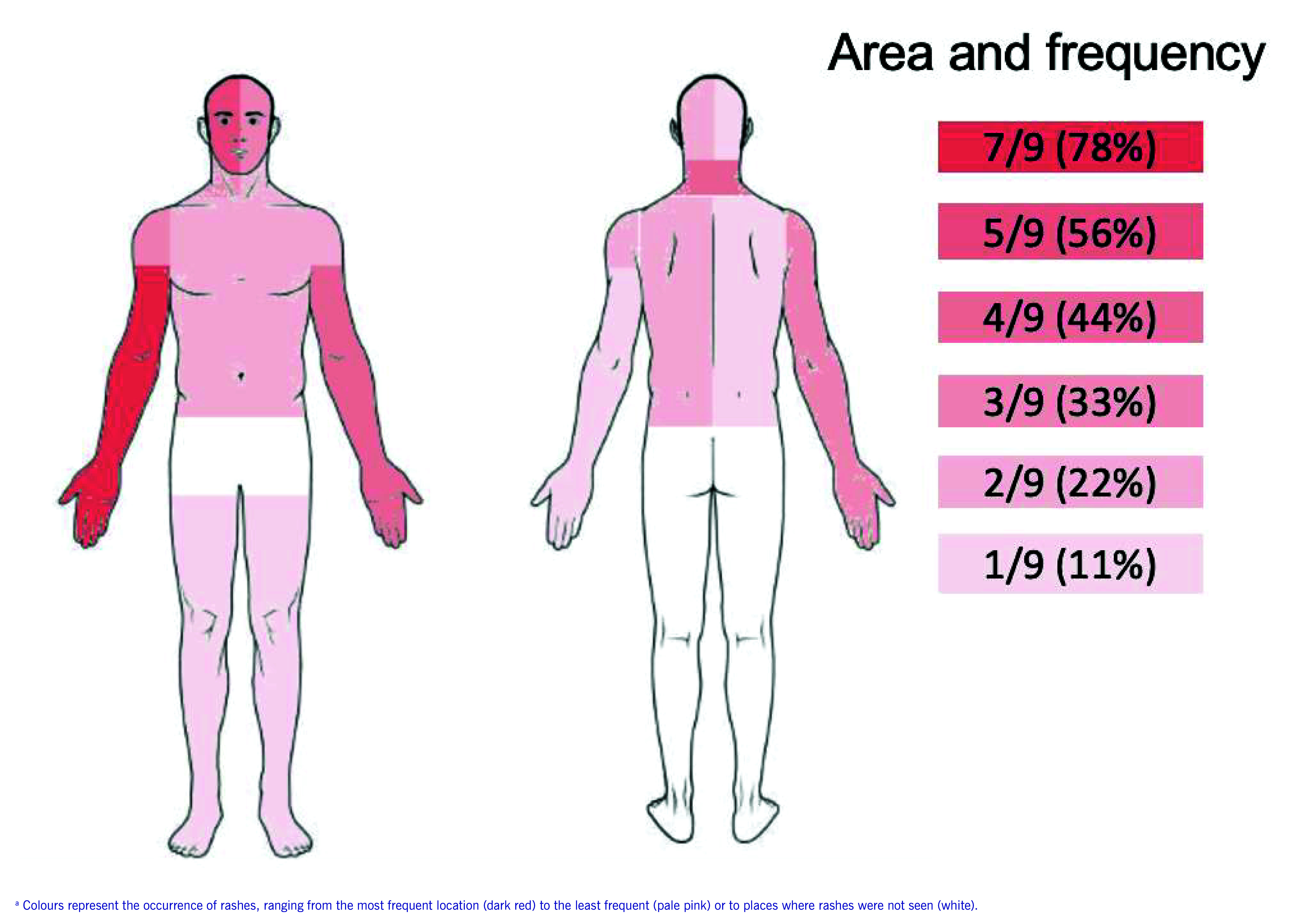
Location of rash among nine confirmed cases of herpes gladiatorum, Phuket, Thailand, May–August 2022

Of the 114 individuals interviewed or examined for rash, 10 had rash symptoms consistent with HG and were classified as suspected cases; nine were tested by RT–PCR to confirm their diagnoses (**Table 1**). One suspected case (a Russian female) was not tested because she left Gym I after the owner prevented her from training. The 10 cases were all non-Thai nationals (seven from Europe, one from New Zealand, one from Russian Federation and one from the United States). The median age was  27 years (interquartile range [IQR]: 21–29 years) (data not shown). All nine confirmed cases were male; one had a history of asthma and another had a prior herpes infection (**Table 1**). The predominant symptom of confirmed cases was a vesicular rash, occurring mainly on the right forearm (7/9 cases, 78%), the right side of the face (5/9, 56%) and the left forearm (4/9, 44%) (**Fig. 1**; **Supplementary Fig. 2**). Additionally, three cases (3/9, 33%) had fever and two (2/9, 22%) had enlarged lymph nodes (data not shown).

[insert Figure 1]

**Table 1:** Characteristics of herpes gladiatorum cases (*n* = 10) and their risk contacts (*n*> = 92) at three Thai-boxing gyms, Phuket, Thailand, May–August 2022

Characteristic	Gym			Total
	I	II	III	
**Cases**				
**Confirmed**	**5**	**3**	**1**	**9**
**Suspected**	**1**	**0**	**0**	**1**
**Sex (male/female)**	**5/1**	**3/0**	**1/0**	**9/1**
**Ethnicity (White/Asian)**	**6/0**	**2/1**	**1/0**	**9/1**
**Country of nationality**	**Ireland 4** **Portugal 1** **Russian Federation 1**	**England 2** **New Zealand 1**	**USA 1**	**10**
**Previous HSV infection**	**0**	**1**	**0**	**1**
**Underlying disease**	**No**	**No**	**1 (asthma)**	**1**
**No. interviewed or examined for rashes/total no.**				
**Owner/manager**	**1/1**	**1/1**	**2/2**	
**Non-Thai trainee**	**18/24**	**12/20**	**21/30**	
**Non-Thai MMA trainee**	**0/0**	**0/21**	**0/50**	
**Thai trainee**	**3/6**	**0/0**	**0/0**	
**Thai trainer**	**6/6**	**10/10**	**10/21**	
**Non-Thai trainer**	**0/0**	**3/3**	**7/7**	
**Thai teenage trainee**	**1/10**	**1/5**	**0/0**	
**Worker**	**0/0**	**6/6**	**12/34**	
**No. interviewed or examined/total no. in gym**	**29/47**	**33/66**	**52/144**	**114/257**
**No. of risk contacts/total no. interviewed or examined^a^**	**28/29**	**26/33**	**38/52**	**92/114**
**Attack rate^b^**				
**Overall**	**21.4% (6/28)**	**11.5% (3/26)**	**2.6% (1/38)**	**10.9% (10/92)**
**Non-Thai trainees**	**33.3% (6/18)**	**25.0% (3/12)**	**4.8% (1/21)**	**19.6% (10/51)**

HSV: herpes simplex virus; MMA: mixed martial arts.

^a^ A risk contact was a person who either had skin-to-skin contact with a suspected or confirmed case, specifically during training or sparring sessions (direct contact), or had used equipment that had been used by a suspected or confirmed case (indirect contact), such as target pads, gloves or punching bags.

^b^ The attack rate was calculated as the number of cases divided by the total number of risk contacts.

#### Case timeline and distribution

**Fig. 2:**
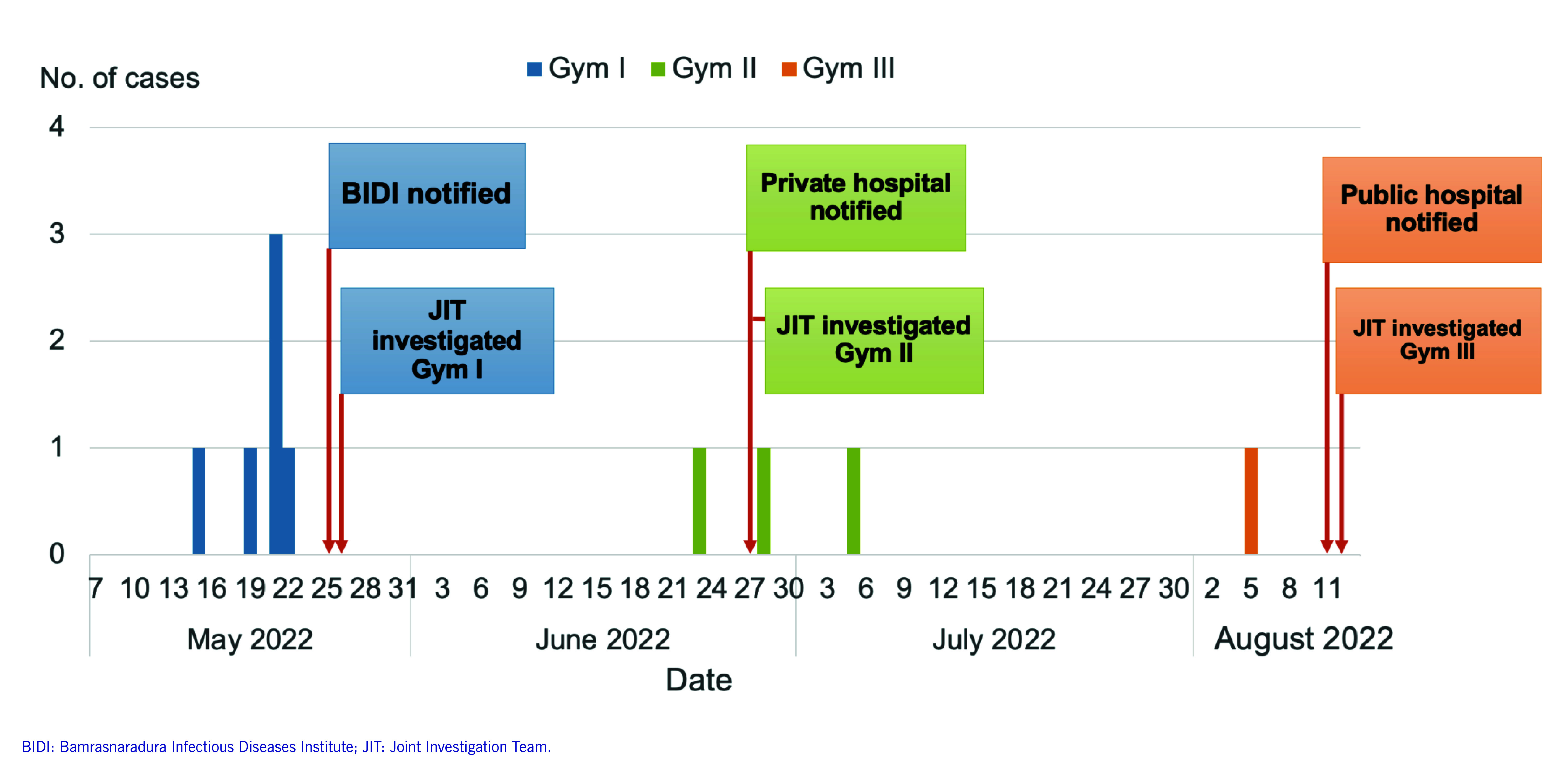
Date of onset of symptoms of herpes gladiatorum at three Thai-boxing gyms, Phuket, May-August 2022 (*N* = 10)

**Fig. 3:**
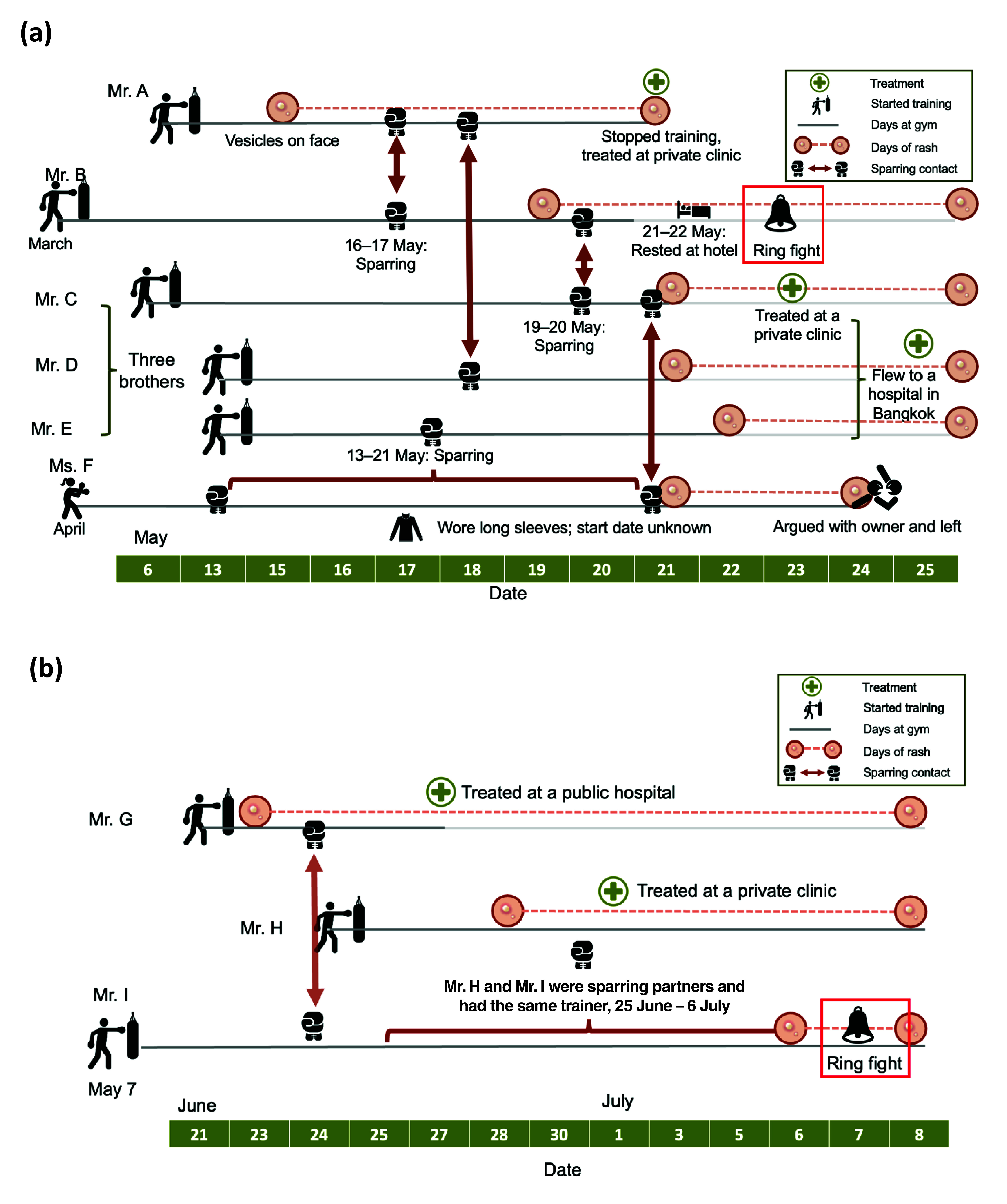
Timeline of cases of herpes gladiatorum in (a) Gym I and (b) Gym II, Phuket, Thailand, May–August 2022

Onset in the first case was 15 May, and the outbreak peaked on 21 May in Gym I, with three cases. Thereafter, Gym II reported sporadic cases and Gym III had one case (**Fig. 2**). A timeline of contacts between confirmed cases and sparring partners at Gyms I and II is shown in **Fig. 3**. It was not possible to construct a meaningful timeline for Gym III, as we were unable to trace many of the 12 known sparring partners of the one suspected case.

[**insert** Figure 2]

Mr A commenced training at Gym I on 6 May and developed facial vesicular rashes by 15 May, marking his exposure period. He continued training with close-contact sparring until 21 May, after which he sought medical treatment. Mr B interacted with Mr A on 16 May. By 19 May, he exhibited a vesicular rash that spread to his neck and caused painful and swollen lymph nodes, leading him to cease training and rest at a hotel on 21–22 May. Financial pressures compelled him to participate in a professional fight on 23 May, at which time he had an open wound on his forehead. Mr C sparred with Mr B on 19 May, and Mr D sparred with Mr A, who had a rash, on 18 May. Mr E sparred with Ms F on 13–21 May; Ms F (the unconfirmed case) also had a rash during this period of time, but Mr E was unaware of her rash because she always wore long-sleeved clothing. Mr C, Mr D and Mr E are brothers, and they all developed rashes that were initially misdiagnosed as bacterial skin infections by a clinic or pharmacy. As their symptoms worsened, they flew to Bangkok for treatment at a private hospital, which subsequently notified BIDI of a suspected mpox cluster. Other trainees noticed large groups of vesicles on Ms F’s body during May, and despite being advised to rest on 24 May, she resisted and departed the gym (**Fig. 3a**).

[**insert** Figure 3]

Three of the nine confirmed cases trained at Gym II, where 33 potential risk contacts were identified. Mr G began training on 21 June and developed groups of vesicles on 23 June. Despite the rash, he sparred with Mr I on 24 June. Mr I developed a rash on 6 July, and the next day he participated in a professional fight for financial reasons. Mr H developed vesicles on 28 June and sparred with Mr I; both were trained by the same Thai trainer (**Fig. 3b**).

Gym III’s single confirmed case (Mr J) began training on 27 June and sparred with a rash-affected partner. His symptoms emerged on 5 August. He then obtained medication from a nearby pharmacy, but it was ineffective. He was hospitalized 2 days later.

At Gym I, six confirmed and suspected cases and 28 risk contacts were interviewed or assessed for rash, yielding an overall attack rate of 21.4% (6/28), rising to 33.3% (6/18) among non-Thai trainees (**Table 1**). The overall attack rates in Gyms II and III were lower, at 11.5% (3/26) and 2.6% (1/38), respectively, but again the rates were elevated among non-Thai trainees, at 25.0% (3/12) and 4.8% (1/21), respectively.

Seven confirmed cases and one trainer were known to have sparred with rash-affected individuals. All trainers and one case shared boxing gloves, and everyone used a shared sandbag. Four cases rarely showered before training, one often did and four always did, while among the trainers, one often showered before training and eight always did (data not shown).

### Environmental study

Gym I comprised a large, roofed and open-sided space (40 × 70 m) with two boxing rings and a smaller enclosed room (10 × 20 m). Sanitation was a concern, with subpar toilet facilities and inconsistent cleaning practices. Boxing gloves, pads and other shared equipment, such as sandbags, leg guards and headgear, were often left uncleaned overnight. Some trainers and trainees resided at an adjacent three-storey apartment complex where the water system was unreliable; this meant that they frequently used the gym’s bathroom facilities.

Gym II was larger, offering a Thai-boxing gym, an MMA gym and a fitness centre, as well as accommodation for trainers and guests. The Thai-boxing gym measured 20 × 25 m, and had two boxing rings and 30 sandbags, and equipment was stored on shelves. The gym area was disinfected twice daily. Accommodation for trainers consisted of eight rooms, each with a bathroom, but cleaning practices varied widely among occupants. The on-site hotel, which also had eight rooms, was cleaned daily.

Gym III, covering 120 × 50 m, was the largest training facility, providing 12 boxing rings, around 50 sandbags, a full-size MMA cage, an indoor weight room, a yoga studio, training areas for MMA and Brazilian jiu-jitsu, and a fitness area. The facility was well maintained, with 34 workers responsible for daily cleaning. However, the insides of gloves and target pads were not cleaned, despite daily use. The on-site hotel, with 10 rooms, was also cleaned daily by the staff.

### Laboratory tests

Specimens were collected from a total of nine suspected cases at all three gyms, while environmental surface samples were obtained only from Gym II. Among the nine confirmed cases, seven were confirmed by positive lesion scrape and three by positive swabs from lesion ulcers. The median Ct value of HSV-1 ranged from 18.7 (IQR: 16.3–26.3) in lesion scrapes to 25.3 (IQR: 24.9–26.9) in throat swabs (data not shown). All samples tested positive for HSV-1; the other pathogens were not detected: HSV-2, mpox virus, varicella–zoster virus and *T. pallidum* (**Table 2**). HSV-1 was found in environmental samples from target pads; a boxing ring floor, and ropes and corners; and an uncleaned sandbag (Ct: 34–36); none were culturable. Water samples from the gym and pool bathrooms showed chlorine levels of 0.25 mg/L; pool water samples contained 2.5 mg chlorine/L.

**Table 2:** Results of laboratory testing of human specimens and environmental samples collected during an investigation of an outbreak of herpes gladiatorum, Phuket, Thailand, May–August 2022

Type of specimen	Result^a^					
	HSV-1	HSV-2	HSV IgG or IgM antibody	Monkeypox virus	Varicella–zoster virus	*Treponema pallidum*
**Human^b^**						
**Throat swab**	**5/5**	**0/2**	**–**	**0/9**	**0/2**	**–**
**Lesion fluid**	**6/6**	**0/3**	**–**	**0/9**	**0/3**	**–**
**Lesion scrape**	**7/7**	**0/5**	**–**	**0/8**	**0/4**	**0/1**
**Lesion ulcer**	**3/3**	**0/3**	**–**	**–**	**0/3**	**–**
**Blood**	**2/3**	**0/2**	**0/3**	**0/6**	**0/2**	**–**
**Environmental surfaces^c^**						
**MMA floor mat**	**0/1**	**–**	**–**	**–**	**–**	**–**
**Fitness floor and equipment**	**0/1**	**–**	**–**	**–**	**–**	**–**
**Target pad**	**1/1**	**–**	**–**	**–**	**–**	**–**
**Boxing ring rope and corner**	**1/1**	**–**	**–**	**–**	**–**	**–**
**Boxing ring floor**	**1/1**	**–**	**–**	**–**	**–**	**–**
**Uncleaned sandbag**	**1/1**	**–**	**–**	**–**	**–**	**–**
**Cleaned sandbag**	**0/1**	**–**	**–**	**–**	**–**	**–**
**Boxing gloves**	**0/1**	**–**	**–**	**–**	**–**	**–**
**Water**	**0/3**	**–**	**–**	**–**	**–**	**–**

HSV: herpes simplex virus; IgG: immunoglobulin G; IgM: immunoglobulin M; MMA: mixed martial arts.

^a^ Results are the number of positive specimens/total number collected. Dashes indicate not applicable or not tested.

^b^ Specimens were collected from the nine confirmed cases.

^c^ Environmental samples were collected only from Gym II.

**Fig. 4:**
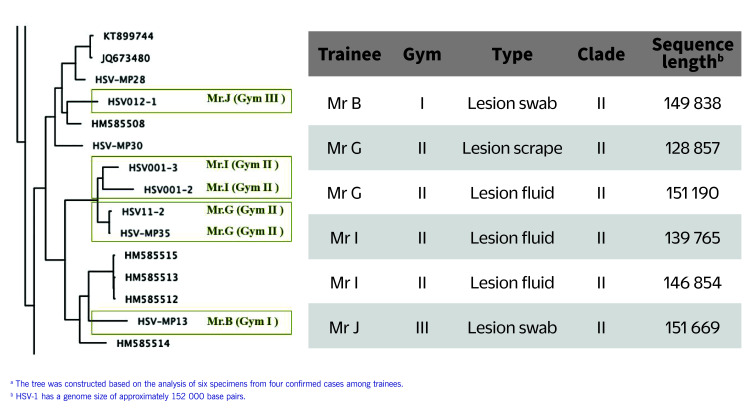
Fig. 4. Phylogenetic tree of confirmed cases of herpes gladiatorum in Phuket, Thailand, May–August 2022^a^

A phylogenetic tree was constructed using data from six specimens obtained from four confirmed cases (**Fig. 4**). All specimens belonged to Clade II, which is the East Asian strain. (*10*) The specimens from Mr G and Mr I, who both trained at Gym II, were found to be in the same phylogenetic node.

[**insert** Figure 4]

## Discussion

We identified nine confirmed and one suspected case of HSV-1 infection in three Thai-boxing gyms in Phuket. The first confirmed case trained at Gym I but had no known prior contact with persons exhibiting rash symptoms before the onset of his own. Identifying the source of his infection is likely to be challenging, as HSV-1 infection can result in people becoming lifelong, asymptomatic carriers. (*7*) Seven of the nine cases had a history of training in sparring and clinching with partners who had a rash, implying transmission through skin contact and an epidemiological link.

The attack rate among non-Thai trainees ranged from 4.8% to 33.3%. In contrast, none of the Thai trainers or trainees were found to have HSV infections. Differences in hygiene habits may have contributed to this discrepancy. While four of the nine non-Thai trainees reported infrequently showering before training, nine out of 10 Thai trainers regularly showered before training. Chlorine levels in the bathrooms met national health standards. Furthermore, four of the nine non-Thai trainees continued to train despite exhibiting symptoms, a behaviour that could facilitate the further spread of infection. However, the lack of data about the showering habits of Thai trainees, along with incomplete information about sparring partners, limited our capacity to fully assess the role of behavioural factors as contributors to higher attack rates in non-Thai trainees.

The distribution of skin rashes and training practices appear to be correlated. The most common areas of the body with a rash were those that made contact during clinching training practices, when trainees use their hands and arms to grasp the back of their opponent’s neck (**Supplementary Fig. 1** and **2**). Most rashes were distributed on the right side of the body. This pattern aligns with the fact that most trainees are right-oriented. A similar observation was made in a report of an HG outbreak among wrestlers in the United States. (*1*)

Two cases with infection confirmed later participated in professional fights while displaying rash symptoms. One of these fighters, who had multiple rashes, had refrained from training in the gym for 2 days before the fight. However, he chose to participate in a fight due to financial constraints. That trainees took part in fights despite having rashes is concerning for two reasons. First, it indicates a lack of enforcement of current Boxing Committee regulations governing pre-fight physical examinations (*12*) and, second, it underscores the economic pressures that can lead fighters to overlook their health.

All three non-Thai trainees whose blood was tested for HSV-1 antibodies were found to have undetectable antibody levels. This finding is consistent with evidence that suggests the prevalence of HSV-1 antibodies is decreasing in high-income countries. (*13*, *14*) In contrast, the Thai trainees generally had lower socioeconomic status, a demographic that has been associated with higher levels of HSV-1 antibodies and a higher degree of immunity to the virus. (*15*) While not definitive, it does seem that differences in socioeconomic status at both the country and individual levels may form part of the explanation for the higher attack rates observed in non-Thai trainees.

Genomic sequencing revealed the presence of the East Asian strain (Clade II), which is the predominant strain within Asia. (*10*) This distribution suggests that the confirmed cases might have contracted the infection in Thailand. Notably, specimens from two trainees from Gym II shared the same phylogenetic node. Since these individuals had engaged in close-contact sparring, epidemiological and genomic evidence suggests a direct link between the two cases. However, cases from the three gyms were grouped on distinct branches of the phylogenetic tree; this means that, although all cases are Clade II, there is no compelling evidence to suggest a direct transmission link between the gyms. It is plausible that the virus was introduced to each independently.

In Gym II, HSV-1 was found on a target pad, a boxing ring rope and an uncleaned sandbag. Despite these items being frequently used by trainers and trainees, especially the universally shared target pad, no rashes were reported among the Thai trainers. The high Ct values and non-culturability of the surface specimens suggested the presence of inactive viral remnants, indicating a lower risk of transmission via these surfaces. Sampling the sandbag before and after cleaning with disinfectant revealed an absence of HSV-1 after cleaning, suggesting that the disinfection practices effectively reduced the risk of transmission from this surface. The specimens from the fitness and MMA sections of the gym were all negative for HSV-1, and these areas were not frequently used by the confirmed cases.

### Public health actions and recommendations

All confirmed cases were seen by doctors at primary care facilities and advised to rest at their accommodation until their rash resolved. Gym owners and trainers were provided with health education and instructions about how to check their skin for signs of infection. The water system at the apartment adjacent to Gym I was repaired.Subsequent to the investigation, the joint investigation team issued guidelines that covered infection control measures, including routine disinfection, personal hygiene and screening for skin lesions before competition. Educational materials about atypical rash diseases were distributed to all Thai-boxing gyms and stadiums in Phuket. In addition, a surveillance system was established to monitor atypical rash diseases in Phuket. Other follow-up actions have included recommending that regulators introduce stricter requirements for pre-fight physical examinations, as well as rules that bar trainees with active infections from participating in training and fights. The joint investigation team also provided recommendations to gym owners, trainers and trainees regarding the need to maintain high standards of hygiene in gyms, including ensuring that boxers shower before training sessions and avoiding sharing equipment, such as boxing gloves and protective gear. Regular cleaning and disinfection of common training areas – such as boxing rings, equipment and mats – were recommended to minimize the risk of surface transmission. Advice was offered to gym owners, staff and trainees to raise awareness about HG and the risk of skin-to-skin infections.

### Limitations

The main limitation of this study is that cases of HG were likely missed, resulting in attack rates that are probably underestimates. Investigations were conducted between 4 and 11 days after onset of the first symptoms, possibly due to an initial misdiagnosis of HG as a bacterial skin infection and consequent delayed notifications. In addition, the relatively short duration of training courses (typically 1–2 weeks) and high turnover rates of trainees limited our ability to trace all potential risk contacts. The owners of Gyms I and III did not permit the team to interview the staff or collect samples from facility surfaces, which limited the investigation.

### Conclusions

Outbreaks of HG were confirmed in three Phuket Thai-boxing gyms. Non-Thai trainees, all of whom came from high-income countries, had the highest attack rates, possibly because of differences in hygiene practices or lower levels of immunity, or both. Genomic analyses suggested local transmission within Thailand, likely through skin-to-skin contact. This study underscores the need for stricter gym hygiene practices, including regular showering before training and not sharing equipment, as well as stricter regulations for pre-fight screening to reduce the risk of transmission of skin infections.
