# Supplementary material for: The first outbreak of herpes gladiatorum in Thailand: an investigation of boxing gyms in Phuket, May–August 2022
Source: Western Pac Surveill Response J. 2025 Dec 1;16(4):1–11. doi: 10.5365/wpsar.2025.16.4.1142 (PMC12820559; doi:10.5365/wpsar.2025.16.4.1142)

Supplementary Fig. 1. **Thai-boxing techniques**

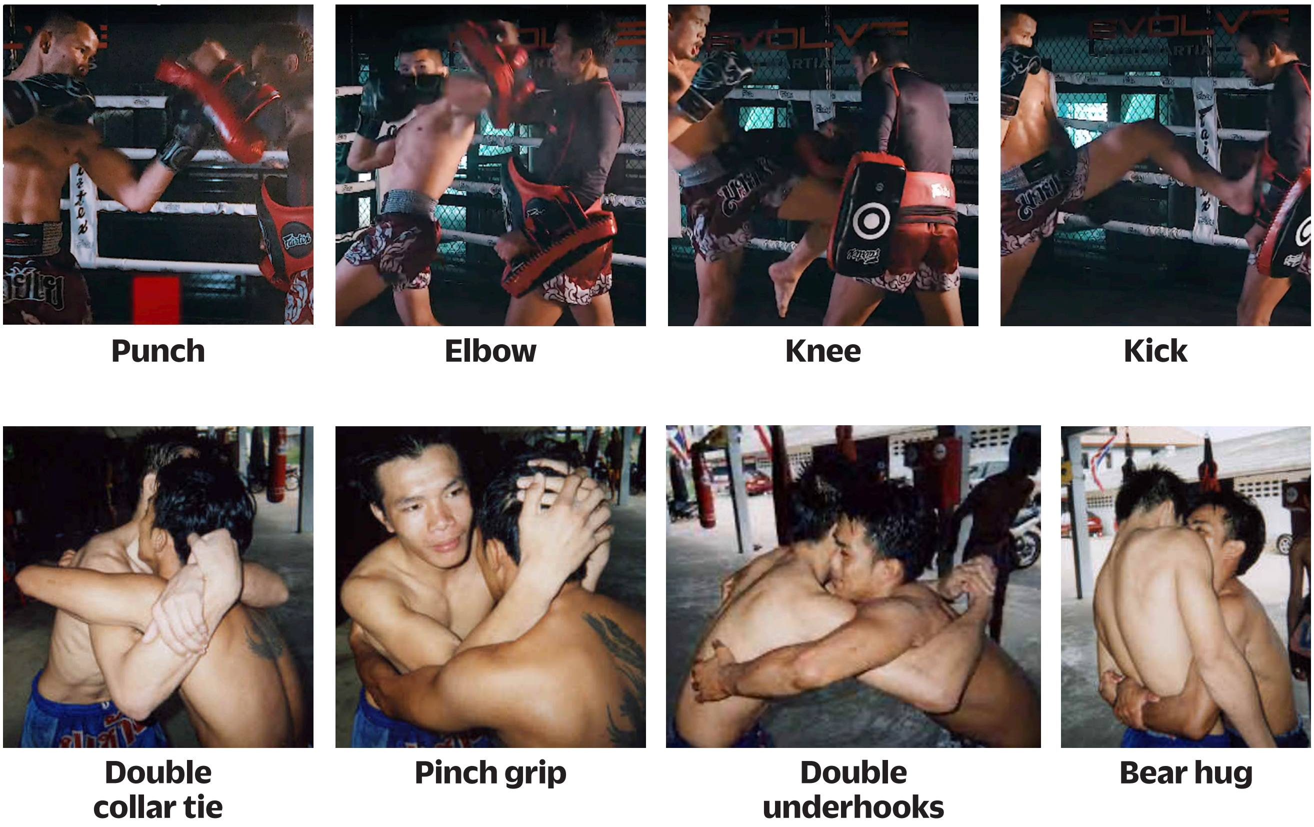

Supplementary Fig. 2. **Characteristic skin rashes among nine confirmed cases of herpes gladiatorum, Phuket, Thailand, 2022**

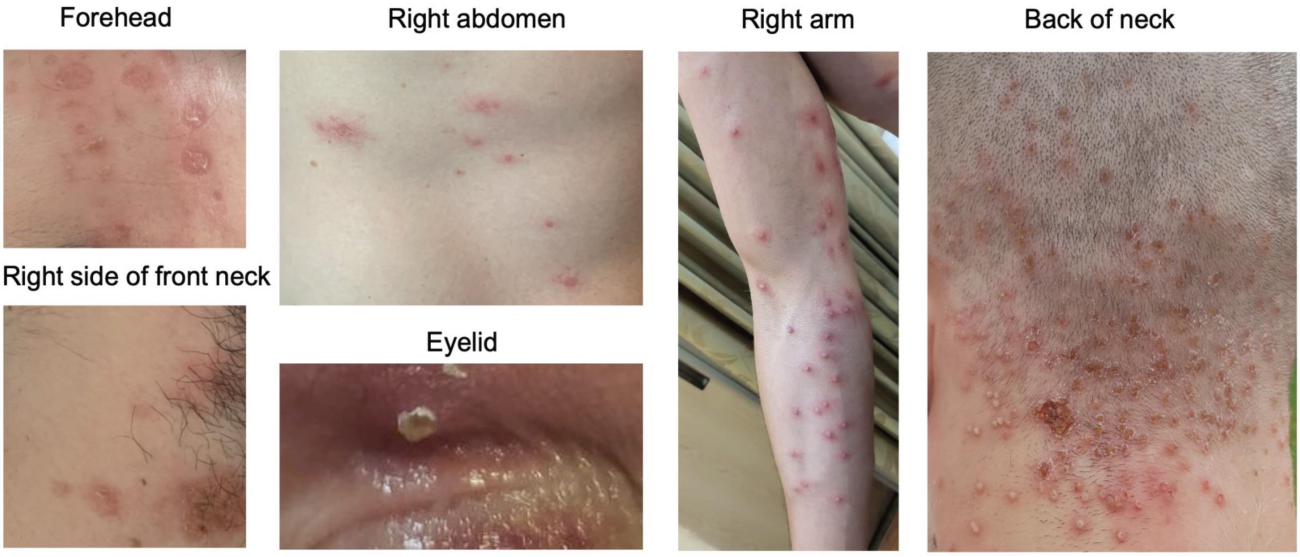

Supplement: Supplementary file 1 [file wpsar-16-1142-s001.pdf]
